# Re-evaluating previous dose and allowing increasing recovery (REPAIR): study protocol for a thoracic reirradiation phase I dose escalation trial

**DOI:** 10.1186/s12885-026-15942-2

**Published:** 2026-04-24

**Authors:** Donna H. Murrell, Nicolaus Andratschke, Alanah M. Bergman, Kristy Brock, Emma M. Dunne, Mitchell Liu, Aliaksandr Karotki, Alexander V. Louie, Andrew Warner, Jason Vickress, X. Melody Qu, David A. Palma

**Affiliations:** 1 Department of Oncology, Schulich School of Medicine & Dentistry, Western University, London, ON Canada; 2 Verspeeten Family Cancer Centre, London Health Sciences Centre, London, ON Canada; 3 University Hospital of Zurich, University Hospital Zurich, Zurich, Switzerland; 4 BC Cancer – Vancouver, Vancouver, BC Canada; 5 The University of Texas MD Anderson Cancer Center, Houston, TX USA; 6 Sunnybrook Health Sciences Centre, Toronto, ON Canada; 7 Department of Radiation Oncology, University of Toronto, Toronto, Canada

**Keywords:** Reirradiation, Lung cancer, Esophageal cancer, Thoracic malignancy, Radiotherapy, Dose escalation

## Abstract

**Background:**

The clinical demand for reirradiation is increasing; however, evidence for dose guidance to estimate normal tissue toxicity is lacking. The goal of the REPAIR trial is to determine the maximally tolerated dose (MTD) of reirradiation in the thorax, implemented by sequentially increasing the normal tissue recovery factors applied to previously delivered dose.

**Methods:**

This multi-institution phase I dose-escalation study for patients undergoing thoracic reirradiation will use a time-to-event continual reassessment method (TITE-CRM). The MTD of reirradiation in this trial is represented by the recovery factor equation associated with a 35% or lower rate of Common Terminology Criteria for Adverse Events (CTCAE) grade 3–5 treatment-related toxicity occurring within 1 year of treatment. Accrual will start at level 1 (recovery factor = 10% at 6 months + 0.75% per month thereafter). Patients (n = 48) will be assigned to recovery factors using the TITE-CRM model. The model will use all available information from previously accrued patients to assign the highest dose with a predicted risk of grade 3–5 toxicity of 35% or less. Patients with recurrence, metastasis, or new primary malignancies in the thorax requiring radiation and who have previously received radiotherapy to the thorax, where reirradiation is expected to exceed the dose constraints used for de novo treatments, are eligible.

**Discussion:**

This study will provide information on the safety of dose escalation for thoracic reirradiation using the concept of recovery after radiation.

**Trial Registration:**

Clinicaltrials.gov identifier: NCT06558175 (registration date August 14, 2024).

## Background

Interest in cancer re-treatment with radiotherapy (reirradiation) is increasing [1, 2]. Reirradiation may be used to treat recurrent, metastatic, or new primary malignancies, and benefits range from palliation to cure. The practice is endorsed by international experts and professional societies [2–6]; however, significant uncertainty remains regarding the safe dose limits in this context.

Prospective clinical evidence to predict toxicity in the setting of reirradiation is lacking. The most comprehensive data exists for re-treatment involving spinal cord where normal tissue tolerance increases by at least 20–25% at 6 months following the first course of radiation [7–11]. Reports also exist for reirradiation in specific sites with defined first and second courses, such as in recurrent head and neck and brain [12]; however, it is hard to translate this data into various re-treatment scenarios involving other organs and different dose/fractionation schemes. Some groups have published cumulative dose constraints for reirradiation based on combinations of literature review, expert opinion, and experience, but these are not based on strong levels of evidence [13–15]. There is an unmet need for prospective clinical trials to determine safe dose limits for reirradiation.

Physicians are left with little guidance on how to perform reirradiation. Safe dose limits are critical because overdosing organs-at-risk (OAR) risks toxicity in patients for whom treatment goals may vary substantially: from symptom relief/prevention to local control, prolonging survival, or even cure. Reirradiation is often dose-limited by concerns over normal tissue toxicity based on the dose limits defined in the de novo setting. Maintaining accumulated dose from current and prior treatments below the de novo OAR dose limits is a safe approach from the perspective of preventing radiation-induced toxicity; however, this is usually not feasible and therefore (sometimes severely) limits the dose that can be offered for reirradiation. For some patients, this means de-escalating to less dose than what they could benefit from and for others this might mean no further radiation is pursued at all. Risk aversion rooted in the principle of non-maleficence, even with inaction may lead to worse outcomes from cancer progression.

Much is still unknown about the magnitude and timeline of radiation injury recovery in most organs; nevertheless, assuming some level of organ recovery after prior radiotherapy and applying forgiveness against previously delivered dose makes reirradiation within standard dose limits feasible. Practice varies widely on application of this principle and implementation becomes more art than science [1, 2, 4]. Several retrospective studies and reviews have described acceptable toxicity profiles with high-dose thoracic reirradiation; however, sufficient information regarding dose metrics and dose accumulation is unavailable, which precludes reproducible implementation in general practice [16–25].

The REPAIR trial endeavors to identify the magnitude of radiation recovery in the thorax and enable safe reirradiation dose escalation. The trial will provide critical information to support shared-decision making and ensure the risk–benefit trade-offs of reirradiation align with each patient’s wishes. In addition, the data from this study will be informative in guiding subsequent studies on the use of reirradiation for other sites (such as the brain, abdomen, and pelvis) and to inform future re-irradiation trials. The objective of this phase I study is to determine the safety of dose escalation via recovery factors applied against previously delivered dose for patients receiving thoracic reirradiation.

## Methods

### Study design

This study will use a time-to-event continual reassessment method (TITE-CRM). The study design is based on previous thoracic dose escalation trials, including RTOG 0813 and SUNSET [26, 27]. Dose will be escalated by allowing increasing amounts recovery (or “forgiveness”) for previously delivered radiation (Table 1).

**Table 1:** Recovery factor equations to be applied against previous dose(s)

Level	Recovery After 6 Months	+ Rate/Month
−1	3%	0.50%
0	10%	0.50%
**1**	**10%**	**0.75%**
2	15%	0.75%
3	15%	1.00%
4	20%	1.00%
5	20%	1.25%
6	25%	1.40%

+ Rate/month is additional recovery for every month thereafter

### Sample size justification and feasibility

In general, TITE-CRM trials require a sample size that is equal to the number of dose levels in the study, multiplied by 6 [28]. In this trial, with 8 dose levels, the sample size will be 48. Given the multi-institutional nature of this study, we estimate that there will be 12–16 patients accrued per year and recruitment will be completed over 36–48 months.

### Objectives

#### Primary endpoints

The primary endpoint of this study is the maximally tolerated dose (MTD) to normal tissues undergoing thoracic reirradiation, implemented by sequentially increasing the normal tissue recovery factors applied to previously delivered dose. The MTD is the recovery factor equation associated with a ≤ 35% rate of grade 3–5 pre-specified treatment-related toxicity occurring within 1 year of treatment.

#### Secondary endpoints

Toxicity, progression of treated disease, distant metastases, progression-free survival, overall survival, patient reported outcomes and quality of life.

### Patient selection

#### Inclusion criteria

1. Pathologically (histologically or cytologically) proven diagnosis of malignancy, with disease in the thorax requiring reirradiation for any treatment intent. This may include primary lung cancer of any type, esophageal cancer, recurrences, and/or metastasis from any primary. The intrathoracic disease at the time of enrollment does not itself require a biopsy if a prior biopsy has been obtained at that location or another body site. If the risk of biopsy is unacceptable and there is no prior confirmation of malignancy even at the time of the initial course of radiation, enrollment is permitted provided that the case is discussed at a multidisciplinary tumor board or peer-review rounds.
2. Must have received prior photon thoracic radiotherapy ≥ 6 months ago
3. Life expectancy > 6 months.
4. Eastern Cooperative Oncology Group (ECOG) performance status 0–2
5. Age ≥ 18 years
6. The current radiation course, when added to the previous radiation doses, exceeds the normal tissue constraints used for de novo treatments for esophagus, heart, lungs, trachea, bronchus, great vessels, or brachial plexus. Forgiveness of the previous dose (i.e. reduction of the previous dose in the cumulative dose calculation) is required to meet constraints. Submission of a pre-plan summary showing the estimated accumulation of current and previously delivered doses is required for registration.

#### Exclusion criteria

1. Persistent toxicity from previously delivered radiation therapy.
2. Prior development of symptomatic radiation pneumonitis or immunotherapy-related pneumonitis from previous treatment, even if resolved.
3. Cumulative radiation dose for all organs-at-risk is already below dose constraints without a recovery factor applied, or with a recovery factor less than the current dose level of the trial. This will be confirmed by the enrolling team after the planning is completed.
4. The reirradiation dose-limiting structure is expected to be spinal cord, chest wall, and/or stomach; this protocol is not meant to study increasing recovery factors for these organs.
5. Any prior thoracic radiotherapy < 6 months ago; OR prior thoracic radiotherapy delivered twice daily (compensation for holiday breaks are permitted), thoracic radiotherapy delivered by brachytherapy, radionuclides, proton beams, or electron beams.
6. Plans for patient to receive daily adaptive radiotherapy in current plan (computed tomography [CT] or magnetic resonance [MR] based).
7. Plans for the patient to receive other local therapy (including standard fractionated radiotherapy and/or surgery) while on this study, except at disease progression.
8. Concurrent systemic therapy (i.e. on the same days as radiation) is not allowed, EXCEPT for patients being treated for intrathoracic lung cancer (non-small cell lung cancer [NSCLC] or small cell lung cancer [SCLC]) with curative intent.
9. For other patients receiving systemic therapy, they are still eligible for enrollment as long as there is a break in systemic therapy during the course of radiation. For example, if a patient has been on palliative pemetrexed and is planning to continue, they can still be enrolled and would continue to receive pemetrexed; reirradiation would be delivered between cycles, possibly requiring a break in systemic therapy. See Sect. 6.9 for further details.
10. Prior surgical intervention that has significantly changed the position of an organ-at-risk (OAR) that is expected to be a dose-limiting structure.
11. Pregnancy
12. The following autoimmune and connective tissue diseases will be excluded: scleroderma and systemic lupus erythematosus.
13. Patients with interstitial lung disease (ILD).

### Pre-treatment evaluation and registration

See Appendix 1 for pre-treatment evaluation.

#### Registration

1. Complete the Enrollment Form in Research Electronic Data Capture (REDCap). Patient Study Number will be generated automatically upon completion of enrollment form in REDCap. Upload the de-identified consent form and signed Eligibility Checklist.
2. Forward the pre-plan showing the total estimated accumulated dose in equivalent dose in 2 Gy fractions (EQD2) to organs-at-risk and dose contributions from each plan using the secure file transfer protocol (SFTP).
  All patients require an uploaded pre-plan to determine eligibility (i.e.to ensure that the model-assigned recovery factor is required to meet dose constraints). To assist with predicting eligibility, the principal investigators (PIs) will communicate the current dose level after every 4 patients enrolled; however, the dose level could change at any time due to newly reported outcomes.
3. Notify the coordinating centre by phone or email that you have completed the enrollment form, uploaded the required documents, and the radiation plans have been sent for review.
4. The coordinating centre will send an email to all sites with a reminder to report any new toxicities for previously enrolled patients, within 24 hours.
5. PI will review the pre-plan and confirm eligibility and assign a dose level. If the patient is eligible the coordinating centre will confirm by email and inform sites of dose level assigned.
6. Site will complete registration by going into REDCap and completing the registration form.
  In situations where the required recovery allowed by the trial (e.g. 25%) is more than required by the oncologist to deliver the prescribed treatment, those patients will not be registeredand they will be removed from the TITE-CRM model.

**Fig. 1:**
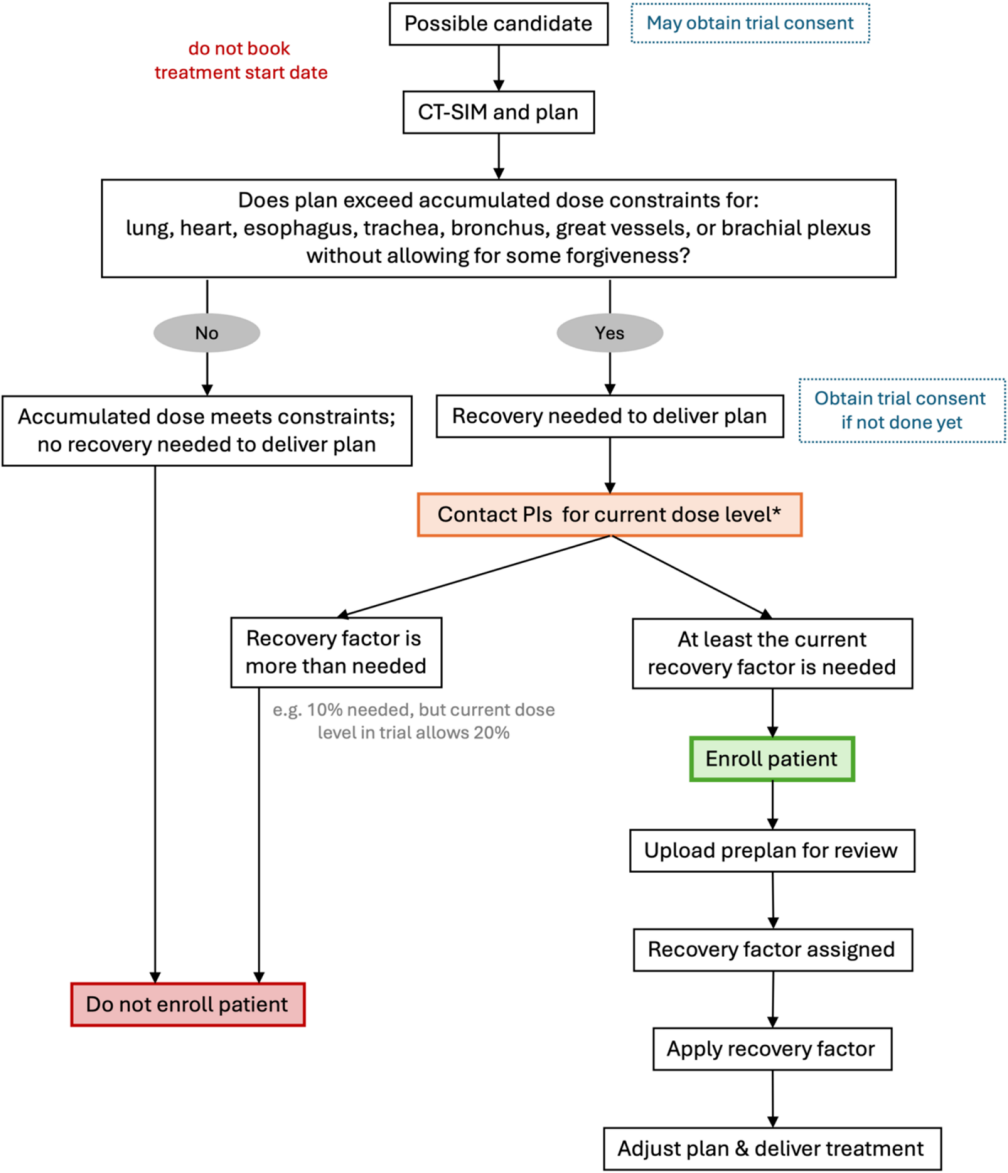
Trial flowchart

The flow chart in Fig. 1 illustrates the process.

#### Example

 The desired current treatment is 30 Gy in 5 fractions to a lung lesion that overlaps the bronchus and will start mid-September 2024. There is expected prescription dose overlap (30 Gy in 5 fractions, 54.0 Gy EQD2/3) at the bronchus with a previous treatment of 60 Gy in 30 fractions, which ended January 31, 2024. In the area of overlap, the bronchus received a prior D0.1 cc = 45 Gy in 30 fractions (40.5 Gy EQD2/3).

Without any forgiveness, the total accumulated near-maximum dose to the bronchus in EQD2 will be: 40.5 EQD2Gy (prior) + 54.0 EQD2Gy (current) = 94.5 EQD2Gy, which is more than the limit of 80 EQD2Gy. This person would require 36% forgiveness against previous dose to meet the constraint.

The study PIs are contacted and note the current trial level is enrolling at Level 4. This would assign a recovery factor of 21% for this case (20% at 6 months plus 1% for an additional 7th month; partial months are not included). This is appropriate and the patient is enrolled. The Level 4 recovery factor is assigned and results in the previous dose contribution decreasing to 32 EQD2Gy. Note the bronchus would still be exceeding the dose constraint (total dose is 86 EQD2Gy) and planning target volume (PTV) would need to be partially compromised so that the bronchus dose is below 28 Gy in 5 fractions (48 EQD2Gy) to meet the 80 EQD2Gy constraint. The plan is adjusted, and treatment is delivered.

#### Optional prospective use of Morfeus

Image registration accuracy is a common challenge in dose accumulation workflows. Morfeus, a biomechanical model-based system, has been shown to reduce uncertainty in deformable image registration for dose accumulation in the thorax [29–32]. This trial will employ optional prospective or retrospective use of Morfeus for deformation of previously delivered dose onto the current planning CT. The turnaround time for this process is anticipated to be 1 business day.

The process for prospective clinical use of Morfeus is outlined below:

- ▪ Anonymized images, structures, and dose files (DICOM format) uploaded to SFTP by local centre.
- ▪ MD Anderson Cancer Centre (MD Anderson) notified and data sent to MD Anderson via SFTP.
- ▪ MD Anderson performs deformable image registration of prior CT to current CT image.
- ▪ MD Anderson to apply deformable image registration to prior dose.
- ▪ MD Anderson to send deformed dose, deformed CT, and deformable image registration deformation vector field (DVF) to London Health Sciences Centre (LHSC) via SFTP.

#### Data collection

- All Serious Adverse Events (SAE) are to be reported to the Central Office within 24 h of discovery. The SAE report is to be completed in REDCap with all available information and Central Office will be notified by phone or email of all SAEs.
- Radiotherapy plan data will be uploaded by SFTP for review.
- All other data will be collected using REDCap, a web based electronic database.

### Radiation Therapy

#### Dose/fractionation

Any prescription dose and fractionation may be included for the current treatment, including stereotactic ablative radiotherapy (SABR). The fractions will be delivered on consecutive weekdays or every second day on weekdays dependent on dose per fraction and institutional standard.

#### Immobilization

Patients will be positioned in a stable position capable of allowing accurate reproducibility of the target position from treatment to treatment. Positions uncomfortable for the patient should be avoided to prevent uncontrolled movement during treatments. Different immobilization systems may be utilized including a thermoplastic shell, alpha-cradle, vac-loc bag or none per institutional standard. The immobilization setup should be identical to that used at simulation time. Each institution will use a consistent technique throughout the study and will inform the trial committee of their approach before enrolling patients.

#### Imaging/motion management

Patients will undergo planning CT simulation with a 4-dimensional CT (4D-CT) capturing the entire lung volume. If institutional standard for motion management, a breath-hold CT simulation would be acceptable. Axial CT images will be obtained throughout the region of interest. At each centre, the local medical physicist or CT-simulation therapists will review the 4D-CT images and will perform the following quality assurance procedures indicated on the 4D-CT template:

i) Ensure all end inspiration (0%) tags exist and are in the right place. This ensures image integrity
ii) The quality of the 4D-CT images should be acceptable (determined by a medical physicist; CT-simulation therapists if standard at that institution).
iii) Motion measurements in all 3 directions are performed.

Motion assessment is mandatory and will be performed via 4D-CT simulation, which is also a required component of this protocol. The specific target motion of each patient must be quantified to determine if additional motion management strategies are required. At a minimum the free breathing internal target volume (ITV) approach will be used for treatment planning and dose-volume histogram (DVH) analysis, although standard institutional management strategies may be used as well. If the tumour excursion is > 1 cm advanced motion management techniques should be considered (e.g. respiratory gating, breath hold, dynamic tumour tracking) if the patient can tolerate.

#### Volume definitions

If the ITV approach is applied, the target lesion will be outlined by a trained physician and designated the gross tumor volume (GTV) on both the inspiratory and expiratory CT images. The target will generally be drawn using CT pulmonary and/or soft tissue windows as appropriate. This target may be enlarged for presumed microscopic extension, at the discretion of the radiation oncologist, and defined as the clinical target volume (CTV). The CTV is optional. Positron emission tomography (PET) images may be used at the discretion of the radiation oncologist.

An ITV is generated from the combined GTV/CTV on the inspiratory and expiratory CT datasets. An additional margin will be added in all planes to constitute the PTV. Institutional PTV margins may be used; in general, these are usually 3–5 mm. Depending on institutional practice, the Exhale phase may be used as a primary dataset for treatment planning with fusion of the inhale, maximum intensity projection (MIP) and average intensity projection. Alternatively, the Average phase may also be used as the primary dataset.

If respiratory gating treatment is used, planning will be performed on a subset average exhale CT dataset (usually labeled either 30%−60% Avg CT or 40%−70% Avg CT). This is an average CT over the intended gated interval. Therefore, the GTV/CTV that is delineated on this scan will incorporate residual motion in the intended gated interval. The PTV for planning will include the GTV (and CTV if used) delineated on the subset average CT and an additional PTV margin (3–5 mm) for setup uncertainty. If breath-hold is used, planning will be performed on the breath-hold CT; targets will be contoured as previously mentioned and a PTV margin applied.

#### Dosimetry

Treatment can be delivered using static beams (either 3D-conformal radiotherapy or intensity-modulated) or rotational therapy (volumetric modulated arc therapy, dynamic conformal arcs or tomotherapy). Single direct beams and parallel-opposed beam set-ups are not allowed. Only photon (x-ray) beams of 10 MV or less energy produced by linear accelerators will be used. Repeat CT and re-plans may be performed for gross changes in anatomy or set-up challenges; however, daily adaptive radiation therapy (ART) and MR linear accelator approaches are not allowed.

For the purposes of dose planning and calculation of monitor units (MUs) for actual treatment, this protocol will require tissue density heterogeneity correction.

Standard institutional dose constraints may be used for the current plan. Cumulative dose constraints listed in Table 2 may not be exceeded; these include both the current dose and the previous dose(s) scaled by the assigned recovery factor(s). Prescription de-escalation and/or target volume compromise is allowed at the discretion of the radiation oncologist to meet current plan and cumulative plan OAR dose constraints. The approach is context-dependent, but in general, this is usually pursued first by compromising PTV coverage, while maintaining GTV coverage, to a minimum dose the radiation oncologist feels to be clinically useful. If that is insufficient, consider decreasing dose and/or increasing number of fractions.

**Table 2:** Organ-at-risk cumulative dose constraints in EQD2. D0.1 cc is the maximum dose in EQD2 allowable to the hottest 0.1 cc. V14.7EQD2Gy refers to the percent of Lung_eval (whole lungs minus all GTVs, trachea/ipsilateral bronchus) receiving 14.7 EQD2Gy or more (and is equivalent to a standard V20 using physical dose)

Contour name	α/β	Metric	Dose Limit
SpinalCanal or SpinalCord_PRV*	2	D0.035 cc	60 EQD2Gy
Brachial_Plexus	3	D0.1 cc	66 EQD2Gy
Esophagus	3	D0.1 cc	70 EQD2Gy
Heart or Heart_eval	3	D0.1 cc	80 EQD2Gy
Trachea	3	D0.1 cc	80 EQD2Gy
BronchialTree	3	D0.1 cc	80 EQD2Gy
GreatVessels	3	D0.1 cc	144 EQD2Gy
Chest Wall	3	D0.1 cc	180 EQD2Gy
Lung_eval	3	V14.7EQD2Gy	37%

^*^Note: recovery factors assigned by the dose level in this trial do not apply to spinal cord. 60 EQD2Gy is allowed irrespective of dose level assigned by the trial. This limit may include 0–20% recovery of previous dose, at the discretion of the radiation oncologist. See example in Note under Evaluation of Accumulated Dose section

Planning OAR volume (PRV) may be used for optimization and to assist with treatment delivery at the discretion of each institution, but dose constraints only apply to the contoured organ itself. Due to the iterative nature of creating new plans and then creating a composite plan, it is unlikely that the OAR doses will reach tolerance exactly. It is recommended that the dose to the dose-limiting OAR is within 3% of this target, without going over, and this data will be captured.

#### Evaluation of accumulated dose

Evaluation of a full 3D dose accumulation in EQD2 is required. Spatially mapping doses has been shown to improve consistency in accumulated dose for reirradiation across multiple institutions [33], therefore dose accumulation must be performed using rigid or deformable image registration. Each previously delivered dose file will be individually registered to the current planning CT. Registration accuracy should be focused on areas of high dose overlap and OARs with cumulative doses near their respective limits. Multiple registrations to evaluate OARs may be necessary. Physical dose will be converted to EQD2 using the α/β ratios defined in Table 2 and the equation below, where D = total dose, and d = dose per fraction.

$$EQD2=D\left(\frac{d+\alpha /\beta }{2+\alpha /\beta }\right)$$

Recovery factors are applied to individual dose distributions after conversion to EQD2. See Table 1 for escalation schemas. Each level includes an initial starting percentage of recovery at 6 months and another small percentage for every month thereafter. The maximum amount of repair is time- and level-dependent, escalating to 100% repair at 5 years in Level 6. Time since previous treatment is calculated based on the last day of each previous radiation course. Always round down when calculating the number of months since previous treatment (e.g. if it has been 6 months and 3 weeks, then count this as 6 months). No further repair will be applied after 5 years, but patients treated more than 5 years since prior radiation are allowed, and the 5-year repair level will be applied. Multiple prior courses of radiation are allowed, but each dose distribution must be scaled by the appropriate recovery factor in Table 1 for each previous course of radiation, depending on when it was delivered.

**Note:** The recovery factor assigned from the trial does not apply to spinal cord. Up to 20% recovery after 6 months is allowed for spinal cord, at the discretion of the radiation oncologist [7, 8], with no further recovery thereafter regardless of the trial assigned recovery factor. For example: if the trial is at Level 1 and it has been 6 months since the previous treatment, 10% recovery will be applied to all organs, but 0–20% can be applied to spinal cord at the discretion of the radiation oncologist. If the trial was at Level 6 and 25% recovery was being applied to all organs, the spinal cord is still limited to 0–20% recovery. See example calculations in Appendix 2, and a list of recovery factors by month in Appendix 3.

Accumulated dose calculations must be reviewed by a radiation oncologist and a medical physicist prior to treatment start. Required components for review are: correct files used, confirmation of delivered (vs. planned) dose and fractions, image registration, EQD2 calculation, dose accumulation, and applied recovery factor.

#### Organs at risk

Organs must be contoured such that appropriate DVHs can be generated. Auto-contouring tools may be used to assist in organ delineation but must be reviewed by a radiation oncologist for definition compliance. Instructions for the contouring of these organs are as follows:

#### Spinal cord

The spinal cord will be contoured based on the bony limits of the spinal canal. The spinal canal should be contoured starting at least 5 cm above the superior extent of the PTV and continuing on every CT slice to at least 5 cm below the inferior extent of the PTV. If the true spinal cord is easily visualized on CT or with a magnetic resonance imaging (MRI), then the spinal cord + 2 mm PRV may be used for this structure instead of the bony limits of the spinal canal. If a very large dose gradient is expected near the spinal cord, then delineating the cord on MRI is recommended. For dosimetry purposes, these structures will be called “SpinalCanal” or “SpinalCord_PRV” respectively.

#### Esophagus

The esophagus will be contoured using mediastinal windowing on CT to correspond to the mucosal, submucosa, and all muscular layers out to the fatty adventitia. The esophagus should be contoured starting at least 5 cm above the superior extent of the PTV and continuing on every CT slice to at least 5 cm below the inferior extent of the PTV.

#### Brachial plexus

The defined ipsilateral brachial plexus originates from the spinal nerves exiting the neuroforamina on the involved side from around C5 to T2. If visualized in the planning CT, the brachial plexus itself should be contoured. However, if not visualized, then for the purposes of this protocol, the major trunks of the brachial plexus may be contoured using the subclavian and axillary vessels as a surrogate for identifying the location of the brachial plexus. This neurovascular complex will be contoured starting proximally at the bifurcation of the brachiocephalic trunk into the jugular/subclavian veins (or carotid/subclavian arteries) and following along the route of the subclavian vein to the axillary vein ending after the neurovascular structures cross the second rib.

#### Heart

The heart will be contoured along with the pericardial sac. The superior aspect (or base) for purposes of contouring will begin at the level of the inferior aspect of the aortic arch (aorto-pulmonary window) and extend inferiorly to the apex of the heart. If the patient is treated in free-breathing, the heart should be contoured on inspiration and expiration images and fused to the average to create a sum named “Heart_eval” for dosimetry purposes.

#### Trachea and proximal bronchial tree

The trachea and proximal bronchial tree will be contoured as two separate structures using mediastinal windows on CT to correspond to the mucosal, submucosa and cartilage rings and airway channels associated with these structures. For this purpose, the trachea will be divided into two sections: the proximal trachea and the distal 2 cm of trachea. The proximal trachea will be contoured as one structure and called “trachea”, and the distal 2 cm of trachea will be included in the structure identified as proximal bronchial tree.

1. Contouring of the proximal trachea should begin at least 5 cm superior to the extent of the PTV or 5 cm superior to the carina (whichever is more superior) and continue inferiorly to the superior aspect of the proximal bronchial tree.
2. The proximal bronchial tree will include the most inferior 2 cm of distal trachea and the proximal airways on both sides. The following airways will be included according to standard anatomic relationships: the distal 2 cm of trachea, the carina, the right and left mainstem bronchi, the right and left upper lobe bronchi, the intermedius bronchus, the right middle lobe bronchus, the lingular bronchus, and the right and left lower lobe bronchi. Contouring of the lobar bronchi will end immediately at the site of a segmental bifurcation.

#### Whole lungs

Both the right and left lungs should be visualized in the planning scan and contoured as one structure. Contouring should be carried out using pulmonary windows. All inflated lung should be contoured; however, gross tumor (GTV) and trachea/ipsilateral bronchus as defined above should not be included in this structure (for dosimetry purposed this may be labeled ‘Lung_eval’).

#### Great vessels

The great vessels (aorta, superior vena cava, pulmonary artery [PA] and pulmonary vein [PV]) will be contoured using mediastinal windowing on CT to correspond to the vascular wall and all muscular layers out to the fatty adventitia. The great vessels should be contoured starting at least 5 cm above the superior extent of the PTV and continuing on every CT slice to at least 5 cm below the inferior extent of the PTV. The distal extent of the right and left PA will be the bifurcation to the basal segmental arteries. The PV will be contoured until the superior and inferior pulmonary veins bifurcate to the segmental and basal veins.

#### Chest wall (for peripheral lesions)

The chest wall will be defined as the 3 cm rind of the ipsilateral hemi-thorax outside the lungs and contoured at least 5 cm superiorly and inferiorly to the PTV.

#### Dose constraints

Table 2 lists near-maximum cumulative dose limits for several critical organs and volume constraints for lung. These are strict limits, and PTV coverage or prescription dose must be adjusted to meet them, if needed. These are mostly based on EQD2 conversions of the UK 2022 Consensus on Normal Tissue Dose-Volume Constraints for 5 fraction treatments [34]. The lung constraint is from RTOG 0617 (V20 in physical dose from the trial is equivalent to 14.7 Gy EQD2Gy) and so is the brachial plexus constraint [35]. The brachial plexus constraint of 60 EQD2Gy in the UK 2022 Consensus was felt to be too conservative because conventionally fractionated lung and head & neck protocols regularly use 66 Gy. There is no clear mandatory constraint for chest wall in the literature. This trial will use a chest wall constraint of 180 EQD2Gy; this is the equivalent of 60 Gy in 5 fractions, which is often allowed in lung SABR where PTV is not compromised for chest wall dose. The focus of this trial is on lung and mediastinal structure doses and escalation based on chest wall alone is not the goal of this protocol. Maximum point dose in undefined normal tissue should be kept as low as reasonably achievable, with a recommended limit of 200 EQD2Gy.

#### Quality assurance

In order to ensure patient safety and effective treatment delivery, a robust quality assurance protocol is incorporated. The following requirements must be completed for each patient:

- Prior to treatment, each patient’s contours, radiation plan, and accumulated dose must be peer-reviewed at the local centre. This can include review by one other radiation oncologist, or discussion at quality assurance (QA) rounds.
- All radiotherapy plans must meet target dose levels for organs at risk (Table 2) after forgiveness is applied to the previous dose. Prior to plan approval, the dose to each OAR must be verified by the medical physicist or radiation oncologist. It is required that dose constraints not be exceeded.
- All dose delivery for intensity-modulated plans (including arc-based treatments) will be confirmed before treatment by physics staff. Patient-specific QA, including MU verification and secondary dose calculations, may be performed by the institutional standard process.
- Online imaging (kilovoltage Cone-beam CT or megavoltage helical CT) will be used to verify patient positioning for each treatment. Ideally, direct tumour localization should be performed. In the absence of direct tumour localization, reliable soft tissue surrogates are recommended.

#### Quality assurance for centres joining study

Prior to opening the study, each participating centre may send up to 5 anonymized test cases via SFTP to ensure treatment plans are designed in compliance with the protocol.

### Systemic therapy

Patients treated with prior systemic therapy are eligible for this study. Cytotoxic agents must.

be held commencing 2 weeks prior to radiation lasting until 1 week after the last fraction, except for curative intent chemoradiation for lung cancers (e.g. NSCLC, SCLC). Molecularly targeted agents must be held for 48 h before the first fraction until 48 h after the last fraction. Immunotherapy and hormone therapy are exempted from these requirements and are allowed during treatment but patients who are on hormone therapy with CDK4/6 inhibitors must stop the latter during treatment. Use of chemotherapy schemes containing potent enhancers of radiation damage (e.g. gemcitabine, doxorubicin) and vascular endothelial growth factor inhibitors (e.g. bevacizumab) are prohibited within the six weeks after radiation.

### Adverse events

Full details of adverse event collection and reporting are provided in Appendix 4. The severity of adverse events will be evaluated using the Common Terminology Criteria for Adverse Events (CTCAE) version 5.0 (http://ctep.cancer.gov).

In the absence of recurrence, any grade 3–5 toxicity listed in Table 3 will automatically be considered to be possibly, probably or definitely related to treatment (i.e. meeting the primary endpoint), unless there is clear evidence that the adverse event is either unrelated or unlikely to be related. The latter instance may occur, for example, in the case of a cardiac event in a patient with an upper lobe tumor and negligible heart dose, or esophageal issues in a patient who received negligible esophageal dose. If a patient starts a drug therapy with overlapping side effects, toxicity may be attributed to the new drug. If there is uncertainty, a final decision will be made by the members of the Data Safety Monitoring Committee (DSMC). See below for causality definitions.

**Table 3:** Examples of treatment related adverse events

Structure	Adverse Event
Cardiac and Pericardial	Constrictive pericarditis
	Symptomatic pericardial effusion
	Pericardial tamponade
	Pericarditis
Gastrointestinal	Esophageal fistula
	Esophageal ulceration
	Esophageal dysmotility
	Esophageal hemorrhage
	Esophageal necrosis
	Esophageal obstruction
	Esophageal stenosis
	Esophageal perforation
Pulmonary/mediastinal	Atelectasis—symptomatic
	Bronchial fistula
	Bronchial obstruction
	Bronchopleural fistula
	Bronchopulmonary hemorrhage
	Dyspnea
	Pneumonitis
	Tracheal/Pulmonary fistula
	Tracheal stenosis
	Mediastinal hemorrhage

In the setting of a recurrence, if a patient develops symptoms of an adverse event but deemed on balance more likely caused by a tumor recurrence than treatment (e.g. pulmonary hemorrhage in the presence of recurrence), this will NOT be considered a treatment-related toxicity.

### Subject discontinuation/withdrawal

Subjects may voluntarily discontinue participation in the study at any time. If a subject is removed from the study, the clinical evaluations that would have been performed at the end of the study should be obtained. If a subject is removed because of an adverse event, they should remain under medical observation as long as deemed appropriate by the radiation oncologist.

### Follow-up evaluation

Patients will be seen in follow-up as outlined in Appendix 1 (every 3 months in year 1, every 6 months in year 2 then annually to 5 years). At each visit, a history and physical examination will be conducted by the oncologist, and CTCAE version 5.0 study treatment related toxicities recorded. Also, the quality-of-life questionnaire will be completed.

CT chest will be repeated at 3, 6, 12, 18 and 24 months then annually to 5 years. Additional imaging or laboratory investigations may be carried out at the discretion of the oncologist. Pulmonary function tests will be repeated at 6, 12 and 24 months then annually. Following progression of disease, future CT scans are optional.

### Statistics and sample size

#### Primary endpoint

The primary endpoint of this study is the MTD of radiotherapy for thoracic reirradiation implemented by sequentially increasing the normal tissue recovery factors applied to previously delivered dose. The MTD is the recovery factor equation associated with a ≤ 35% rate of grade 3-5 treatment-related toxicity occurring within 1 year of treatment. Refer to Table 3 for examples of treatment related toxicities.

#### Secondary endpoints

- Toxicity
- Progression of treated disease,
- Time to distant metastases
- Progression-free survival,
- Overall survival
- Patient-reported outcomes and quality of life: using the Functional Assessment of Cancer Therapy: Lung (FACT-L) and the EuroQol 5-dimension 5-level (EQ-5D-5L)

#### Statistical design

This study will use a time-to-event continual reassessment method (TITE-CRM). The study design is based on RTOG 0813 and SUNSET trials.

#### Recovery factor levels

Recovery factor levels are described in Tables 1 and 4.

**Table 4:** Recovery factor levels

Level	Recovery After 6 Months	Rate/Month	Recovery After 1 Year	Recovery After 3 Years	Recovery After 5 Years	Estimated risk of DLT for initial TITE-CRM model*
−1	3%	+ 0.50%/m	6%	18%	30%	0.034
0	10%	+ 0.50%/m	13%	25%	37%	0.048
1	10%	+ 0.75%/m	15%	33%	51%	0.067
2	15%	+ 0.75%/m	20%	38%	56%	0.094
3	15%	+ 1.00%/m	21%	45%	69%	0.130
4	20%	+ 1.00%/m	26%	50%	74%	0.181
5	20%	+ 1.25%/m	28%	58%	88%	0.252
6	25%	+ 1.40%/m	33%	67%	100%	0.350

Rate/month is additional recovery for every month thereafter

#### Dose assignment procedure

Accrual will start at Level 1 (recovery factor = 10% at 6 months + 0.75% per month thereafter). This recovery factor equation was selected as it translates to approximately 10%/year, which is used in common practice in many Canadian centres, and a lower starting dose may compromise local control for patients enrolled initially. The maximum dose level (Level 6, with 100% recovery at 5 years) was chosen as several studies have reported full-dose thoracic reirradiation. Further dose escalation beyond Level 6 may be investigated in future studies when more is known about recovery time dynamics and organ-specific tissue responses.

Patients will be assigned to recovery factors using the TITE-CRM model (Table 4). The model will use all available information from previously accrued patients to assign the highest dose with a predicted risk of grade 3 toxicity of 35% or less (a similar allowable toxicity level to phase I trials using 3 + 3 designs), with the additional restrictions outlined below.

In the TITE-CRM design, to allow the trial to remain open to accrual without interruption, and in order to use the maximum information from patients already accrued when assigning dose levels, data from patients who have not completed the 1-year follow-up period are weighted according to the proportion of follow-up completed. Weights will range from 0 to 1, with zero denoting no follow-up and 1 denoting 12 months of follow-up (e.g. a patient with 6 months of follow-up will be weighted as 0.5). Any patient with a grade 3–5 toxicity will be weighted as 1, regardless of length of follow-up.

The following restrictions on dose escalation will also be employed:

- Dose levels may only increase one level between consecutive patients;
- A patient may not be assigned to a higher dose level unless there is at least 2 years of cumulative observations on patients at the current dose level. This cumulative observation may be divided among several patients (e.g. four patients treated on Level 1 and followed for 6 months each would constitute 24 months of cumulative observation).

In situations where the required recovery allowed by the trial (e.g. 25%) is more than required by the oncologist to deliver the prescribed treatment, those patients will not be enrolled**.**

#### Sample size

In general, TITE-CRM trials require a sample size that is equal to the number of dose levels in the study, multiplied by 6. In this scenario, the sample size will be 48 [28].

#### Statistical analysis

Descriptive statistics will be generated for baseline patient characteristics for all enrolled patients (*n* = 48). Adverse events attributed to treatment will be reported using frequencies and percentages, as maximum reported grade per patient (including proportion of patients with grade ≥ 2 and grade ≥ 3) and separately by individual adverse event categories. Time-to-event endpoints will be estimated using the Kaplan–Meier method for all patients. Median follow-up will be calculated using the reverse Kaplan–Meier method. Quality of life measured using FACT-L scores and utilities measured using the EQ-5D-5L will be stratified by follow-up visit and compared with baseline data using the paired t-test or Wilcoxon signed rank test (in the event these data are non-normally distributed), as appropriate. Linear mixed modelling will be performed to test for changes in quality of life over time, with time as a fixed effect (modelled as continuous) and adjusting for patient number as a random effect (without interaction). Univariable and multivariable Cox proportional hazards and logistic regression will be performed to identify factors predictive of time-to-event endpoints and adverse events of grade ≥ 2 attributed to treatment, respectively. All statistical analysis will be performed using SAS version 9.4 software (SAS Institute, Cary, NC, USA), using 2-sided statistical testing at the 0.05 significance level.

### Data safety monitoring committee (DSMC)

The DSMC will evaluate AEs on a semi-annual basis. The TITE-CRM model will ensure that the risk of grade 3–5 toxicity doesn’t exceed 35%. However, the DSMC will monitor the risk of related grade 5 toxicity occurring at any time within 1 year of treatment and will track all grade 5 toxicities reported within the 5 year follow-up period. If any dose level sustains 3 related grade 5 toxicities within the first 10 patients on that level, that dose level (and higher) will be closed.

## Discussion

The increasing use of reirradiation in cancer care is likely a result of several underlying factors, including the improved longevity of cancer patients, the availability of better technologies to detect recurrence, the increased conformality of radiation planning, and the development of highly precise radiation delivery techniques [1, 2]. However, high-quality clinical evidence to support reirradiation is sparse, and the optimal reirradiation doses are unknown. When delivering radiation in a previously irradiated area, the treating team must balance two competing priorities: the need to maximize the amount of radiation delivered to tumor (to prevent or delay cancer-related morbidity and mortality) vs. the need to minimize the radiation dose to normal tissues (to avoid causing harm). To balance these trade-offs, a better understanding of the tolerance of thoracic organs to reirradiation is urgently needed. REPAIR will address this need by systematically and carefully increasing the allowable radiation recovery factor, with the goal of allowing the delivery of higher doses to tumor while keeping toxicity rates low.

The TITE-CRM trial design is well-suited to dose-escalation when long-term toxicities are a concern. Standard phase I dose escalation trials use a 3 + 3 design, particularly in the setting of systemic therapy, which delivers the treatment to 3 patients, and then pauses accrual to allow time to observe those patients for toxicities of interested before escalating the dose in the next cohort. While this works well for short-term toxicities, treatments that are associated with long-term toxicities are not well-suited to the 3 + 3 design, since long pauses in accrual would be required. TITE-CRM is a model-based escalation protocol that keeps accrual open without pauses during the trial, uses the information from previously enrolled patients to calculate toxicity probabilities, which allows phase I radiation trials to be completed with a modest sample size in relative short periods of time [36].

There are several important limitations to REPAIR. First, there are uncertainties in dose accumulation, and variability can occur when using different image registration techniques [33]. We will attempt to understand this variability using central collection of plans for analysis, and will mitigate this variability by requiring completion of test plans at each site prior to accrual, and by offering the use of Morfeus image registration technology as part of the trial. Second, REPAIR applies a single recovery factor to all organs, but individual organs may differ in their capacity for radiation repair. This recovery factor is not adjusted for other factors known to predispose to toxicity, such as diabetes and immunosuppression. More advanced models are required, and will likely be developed, to account for individual organ recover while accounting for other risk factors. Finally, although REPAIR can compare outcomes for patients across the different dose levels within the trial, ideally randomized trials of reirradiation will be conducted to best assess the benefits of reirradiation at different levels of recovery.

In summary, the REPAIR trial will use a TITE-CRM dose escalation technique to determine the maximum allowable recovery of normal tissues in the reirradiation setting. The results are expected to assist physicians, physicists and dosimetrists in optimizing the balance between tumor control and risks of toxicity, to ultimately minimize both cancer- and treatment-related morbidity and mortality for patients undergoing reirradiation.

## Data Availability

Not Applicable.

## Appendix 1

**Schedule of Assessments** All times are measured from date of enrollment. Clinical follow-up visits can be ± 6 weeks of the stated time points, scans should be ± 3 weeks

**Table:**

	**Before** **Treatment**	**Year 1**	**Year 2**	**Year 3–5**
History and Physical examination	X*	3, 6, 9, 12 mo	18 mo, 24 mo	36, 48, 60 mo
CT chest	X	3, 6, 12 mo	18, 24 mo	36, 48, 60 mo
Pulmonary Function Testing	X	6,12 mo	24 mo	36, 48, 60 mo
Adverse Event Assessment	X	3, 6, 9, 12 mo	18, 24 mo	36, 48, 60 mo
FACT-L and EQ-5D-5L QOL scoring	X	3, 6, 9, 12 mo	18, 24 mo	36, 48, 60 mo

*Abbreviations*: *mo *months

*including weight and extent of weight loss

## Appendix 2

Example calculations for recovery factor

**Table:**

Assigned Level	Time since Previous Treatment	Recovery Factor Calculation
Level 1	6 months	10 + 0 = 10%
Level 1	1 year	10 + [0.75% * (6 months)] = 14.5%﻿
Level 1	1 year and 3 weeks	Partial months not included; round down Recovery same as above, 14.5%
Level 1	10 years	No further repair after 5 years; apply 5 years for calculation 10 + [0.75% * ({5 years * 12 months/year} – 6 months)] = 50.5%^†^
Level 3	Two prior courses: Course 1 = 3 years ago Course 2 = 2 years ago	For course 1: 15 + [1% * ({3 years * 12 months/year} – 6 months)] = 45% For course 2: 15 + [1% * ({2 years * 12 months/year} – 6 months)] = 33%

^†^in practice, recovery values will be rounded to the nearest whole number so 14.5￫15% and 50.5￫51%

Example calculation for proximal bronchial tree dose at Level 1

**Table:**

**Cou****rse**	**Physical Dose**	**EQD2****G****y**	**Recovery**	**Calculation**	**EQD2****G****y** **Applied to Cumulative Plan**
1 (previous course 1 year ago)	60Gy/30	60	15% (see calculation above)	60*0.85	51
2 (current course)	30Gy/10	36	0%	N/A	36
Total					87 < 88 EQD2Gy

*note point dose calculations are not sufficient for this trial; full 3D EQD2 dose evaluation based on dose distribution is required; the table above is for illustrative purposes of the dose scaling and dose constraint only

## Appendix 3

Calculated recovery factors per month

**Table:**

Months	Level −1	Level 0	Level 1	Level 2	Level 3	Level 4	Level 5	Level 6
1	N/A	N/A	N/A	N/A	N/A	N/A	N/A	N/A
2	N/A	N/A	N/A	N/A	N/A	N/A	N/A	N/A
3	N/A	N/A	N/A	N/A	N/A	N/A	N/A	N/A
4	N/A	N/A	N/A	N/A	N/A	N/A	N/A	N/A
5	N/A	N/A	N/A	N/A	N/A	N/A	N/A	N/A
6	3%	10%	10%	15%	15%	20%	20%	25%
7	4%	11%	11%	16%	16%	21%	21%	26%
8	4%	11%	12%	17%	17%	22%	23%	28%
9	5%	12%	12%	17%	18%	23%	24%	29%
10	5%	12%	13%	18%	19%	24%	25%	31%
11	6%	13%	14%	19%	20%	25%	26%	32%
**12**	**6%**	**13%**	**15%**	**20%**	**21%**	**26%**	**28%**	**33%**
13	7%	14%	15%	20%	22%	27%	29%	35%
14	7%	14%	16%	21%	23%	28%	30%	36%
15	8%	15%	17%	22%	24%	29%	31%	38%
16	8%	15%	18%	23%	25%	30%	33%	39%
17	9%	16%	18%	23%	26%	31%	34%	40%
18	9%	16%	19%	24%	27%	32%	35%	42%
19	10%	17%	20%	25%	28%	33%	36%	43%
20	10%	17%	21%	26%	29%	34%	38%	45%
21	11%	18%	21%	26%	30%	35%	39%	46%
22	11%	18%	22%	27%	31%	36%	40%	47%
23	12%	19%	23%	28%	32%	37%	41%	49%
**24**	**12%**	**19%**	**24%**	**29%**	**33%**	**38%**	**43%**	**50%**
25	13%	20%	24%	29%	34%	39%	44%	52%
26	13%	20%	25%	30%	35%	40%	45%	53%
27	14%	21%	26%	31%	36%	41%	46%	54%
28	14%	21%	27%	32%	37%	42%	48%	56%
29	15%	22%	27%	32%	38%	43%	49%	57%
30	15%	22%	28%	33%	39%	44%	50%	59%
31	16%	23%	29%	34%	40%	45%	51%	60%
32	16%	23%	30%	35%	41%	46%	53%	61%
33	17%	24%	30%	35%	42%	47%	54%	63%
34	17%	24%	31%	36%	43%	48%	55%	64%
35	18%	25%	32%	37%	44%	49%	56%	66%
**36**	**18%**	**25%**	**33%**	**38%**	**45%**	**50%**	**58%**	**67%**
37	19%	26%	33%	38%	46%	51%	59%	68%
38	19%	26%	34%	39%	47%	52%	60%	70%
39	20%	27%	35%	40%	48%	53%	61%	71%
40	20%	27%	36%	41%	49%	54%	63%	73%
41	21%	28%	36%	41%	50%	55%	64%	74%
42	21%	28%	37%	42%	51%	56%	65%	75%
43	22%	29%	38%	43%	52%	57%	66%	77%
44	22%	29%	39%	44%	53%	58%	68%	78%
45	23%	30%	39%	44%	54%	59%	69%	80%
46	23%	30%	40%	45%	55%	60%	70%	81%
47	24%	31%	41%	46%	56%	61%	71%	82%
**48**	**24%**	**31%**	**42%**	**47%**	**57%**	**62%**	**73%**	**84%**
49	25%	32%	42%	47%	58%	63%	74%	85%
50	25%	32%	43%	48%	59%	64%	75%	87%
51	26%	33%	44%	49%	60%	65%	76%	88%
52	26%	33%	45%	50%	61%	66%	78%	89%
53	27%	34%	45%	50%	62%	67%	79%	91%
54	27%	34%	46%	51%	63%	68%	80%	92%
55	28%	35%	47%	52%	64%	69%	81%	94%
56	28%	35%	48%	53%	65%	70%	83%	95%
57	29%	36%	48%	53%	66%	71%	84%	96%
58	29%	36%	49%	54%	67%	72%	85%	98%
59	30%	37%	50%	55%	68%	73%	86%	99%
**60**	**30%**	**37%**	**51%**	**56%**	**69%**	**74%**	**88%**	**100%**

## Appendix 4

### Adverse event definition and reporting

An adverse event (AE) or reaction is any unfavorable and unintended sign (including an abnormal laboratory finding), symptom, or disease temporally associated with the use of a medical treatment or procedure that may or may not be considered related to the medical treatment or procedure.

A serious adverse event (SAE)or reactionas defined in the ICH Guideline: Clinical Safety Data Management: Definitions and Standards for Expedited Reporting, E2A Section IIB includes any untoward medical occurrence that at any dose:

- Results in death
- Is life-threatening (refers to an event in which the patient was at risk of death at the time of the event; it does not refer to an event which hypothetically might have caused death if it were more severe.)
- Results in persistent or significant disability/incapacity
- Requires in-patient hospitalization or prolongation of existing hospitalization
- Is a congenital anomaly/birth defect

Important medical events that may not be immediately life-threatening or result in death or hospitalization may be considered a serious adverse event, when, based upon medical and scientific judgment, they may jeopardize the patient or may require intervention to prevent one of the other outcomes listed in the definition above. Unexpected adverse reaction is one that the nature and severity is not consistent with the applicable product information (e.g., Investigator’s Brochure or Product Monograph, described in the REB/IRB approved research protocol or informed consent document), or occurs with more than expected frequency.

All AEs will be collected starting at baseline. There is no time-limit for collection of AEs and SAEs after treatment within the 5-year follow-up period (e.g. a treatment-related complication at four years post-treatment will still be captured). All AEs are to be captured during treatment and only study-related AEs in the follow-up period.

#### Radiation therapy adverse events

The rationale for this study is to determine the MTD for thoracic reirradiation implemented by increasing the amount of forgiveness applied to previously delivered dose. There is concern about RT effects on organs at risk, most notably central airway, bronchioles/alveoli, esophagus, pulmonary vessels, and heart/pericardium. It is currently unknown how different combinations of conventional and/or SBRT doses may interact to produce toxicity.

If there are baseline symptoms, such as grade 2 dyspnea, a toxicity will only be counted if the grade increases during follow up (for example: if the patient then develops grade 3 dyspnea, then it is counted as a grade 3 event, but if its stays as a grade 2 then it is not counted as a toxicity).

#### Causality (attribution)

In the absence of recurrence, any Grade 3-5 toxicity listed below will automatically be considered to be possibly, probably or definitely related to treatment (i.e. meeting the primary endpoint), unless there is clear evidence that the adverse event is either unrelated or unlikely to be related. The latter instance may occur, for example, in the case of a cardiac event in a patient with an upper lobe tumor and negligible heart dose, or esophageal issues in a patient who received negligible esophageal dose. If a patient starts a drug therapy with overlapping side effects, toxicity may be attributed to the new drug. If there is uncertainty, a final decision will be made by the members of the Date Safety Monitoring Committee (DSMC). See below for causality definitions.

In the setting of a recurrence, if a patient develops symptoms of an adverse event but deemed on balance more likely caused by a tumor recurrence than treatment (e.g. pulmonary hemorrhage in the presence of recurrence), this will NOT be considered a treatment-related toxicity.

An adverse event or reaction is considered related to the research intervention (i.e. radiotherapy) if there is a reasonable possibility that the reaction or event may have been caused by the research intervention (i.e. a causal relationship between the reaction and the research intervention cannot be ruled out by the investigator(s)). The relationship of an AE to the study treatment (causality) will be described using the following definitions:

- Unrelated: Any adverse event for which there is evidence that an alternative etiology exists or for which no timely relationship exists to the administration of the study treatment and the adverse event does not follow any previously documented pattern. The adverse event, after careful consideration by the investigator, is clearly and incontrovertibly due to causes other than the intervention.
- Unlikely: Any adverse event for which the time relationship between the study treatment and the event suggests that a causal relationship is unlikely and/or the event is more likely due to the subject’s clinical condition, or other therapies concomitantly administered to the subject.
- Possible: Any adverse event occurring in a timely manner after the administration of the study treatment that follows a known pattern to the intervention and for which no other explanation is known. The adverse event, after careful consideration by the investigator, is considered to be unlikely related but cannot be ruled out with certainty.
- Probable: Any adverse event occurring in a timely manner after the administration of the study treatment that follows a known pattern to the intervention and for which no other explanation is known. The adverse event, after careful consideration by the investigator, is believed with a high degree of certainty to be related to the intervention
- Definitely Related: Any adverse event occurring within a timely manner after administration of the study treatment that is a known sequela of the intervention and follows a previously documented pattern but for which no other explanation is known. The adverse event is believed by the investigator to be incontrovertibly related to the intervention.

#### Severity

The severity of adverse events will be evaluated using the Common Terminology Criteria for Adverse Events (CTCAE) v5.0 grading scale (see (http://ctep.cancer.gov).

- Grade 1: Mild
- Grade 2: Moderate
- Grade 3: Severe
- Grade 4: Life-threatening or disabling
- Grade 5: Death

#### Immediately reportable adverse events

Any grade 3 to 5 adverse reaction that is definitely, probably, or possibly the result of experimental treatment (i.e. radiotherapy) must be verbally reported to the Principal Investigator and Coordinating Centre and entered into the REDCap database AE log within 1 business day of discovery. This is important due to the nature of the CRM model. Such events must be reported to the approving research ethics board (REB) as per their reporting guidelines.

Conditions that are not related to study treatment are not considered a SAE in this protocol (e.g. hospitalization for routine procedures, or disease progression)

## Abbreviations

4D-CT: 4-Dimensional CT
ART: Adaptive radiation therapy
CT: Computed tomography
CTCAE: Common Terminology Criteria for Adverse Events
CTV: Clinical target volume
DSMC: Date Safety Monitoring Committee
DVF: Deformation vector field
DVH: Dose-volume histogram
ECOG: Eastern Cooperative Oncology Group
EQ-5D-5L: EuroQol 5-dimension 5-level
EQD2: Equivalent dose in 2 Gy fractions
EQD3: Equivalent dose in 3 Gy fractions
FACT-L: Functional Assessment of Cancer Therapy: Lung
GTV: Gross tumor volume
ILD: Interstitial lung disease
ITV: Internal target volume
LHSC: London Health Sciences Centre
MIP: Maximum intensity projection
MR: Magnetic resonance
MRI: Magnetic resonance imaging
MTD: Maximally tolerated dose
MU: Monitor unit
NSCLC: Non-small cell lung cancer
OAR: Organ-at-risk
PA: Pulmonary artery
PET: Positron emission tomography
PI: Principal investigator
PRV: Planning organ-at-risk volume
PTV: Planning target volume
PV: Pulmonary vein
QA: Quality assurance
REDCap: Research Electronic Data Capture
REPAIR: Re-evaluating previous dose and allowing increasing recovery
SABR: Stereotactic ablative radiotherapy
SAE: Serious adverse event
SCLC: Small cell lung cancer
SFTP: Secure file transfer protocol
TITE-CRM: Time-to-event continual reassessment method

## References

[1] Willmann J, Appelt L, Balermpas P, Baumert G, de Ruysscher D, Hoyer M, et al. Re-irradiation in clinical practice: Results of an international patterns of care survey within the framework of the ESTRO-EORTC E2-RADIatE platform. Radiotherapy and Oncology. 2023;189. 10.1016/j.radonc.2023.109947. 10.1016/j.radonc.2023.109947 37806559

[2] Andratschke N, Willmann J, Phd T-L, Leeds ) ;, Andratschke N, Willmann J, et al. European Society for Radiotherapy and Oncology and European Organisation for Research and Treatment of Cancer consensus on re-irradiation: definition, reporting, and clinical decision making. Lancet Oncology. 2022;23:469–78. 10.1016/S1470-2045(22)00447-8 36174633

[3] Ng WT, Soong YL, Ahn YC, AlHussain H, Choi HCW, Corry J, et al. International recommendations on reirradiation by intensity modulated radiation therapy for locally recurrent nasopharyngeal carcinoma. Int J Radiat Oncol Biol Phys. 2021;110:682–95. 10.1016/j.ijrobp.2021.01.041. 33571626 10.1016/j.ijrobp.2021.01.041

[4] Rulach R, Ball D, Chua KLM, Dahele M, De Ruysscher D, Franks K, et al. An International Expert Survey on the Indications and Practice of Radical Thoracic Reirradiation for Non-Small Cell Lung Cancer. Adv Radiat Oncol. 2021;6. 10.1016/j.adro.2021.100653. 10.1016/j.adro.2021.100653 PMC8022147 33851065

[5] Slevin F, Aitken K, Alongi F, Arcangeli S, Chadwick E, Chang AR, et al. An international Delphi consensus for pelvic stereotactic ablative radiotherapy re-irradiation. Radiother Oncol. 2021;164:104–14. 10.1016/j.radonc.2021.09.010. 34560186 10.1016/j.radonc.2021.09.010

[6] Jereczek-Fossa BA, Marvaso G, Zaffaroni M, Gugliandolo SG, Zerini D, Corso F, et al. Salvage stereotactic body radiotherapy (SBRT) for intraprostatic relapse after prostate cancer radiotherapy: An ESTRO ACROP Delphi consensus. Cancer Treatment Reviews. 2021;98. 10.1016/j.ctrv.2021.102206. 10.1016/j.ctrv.2021.102206 33965893

[7] Nieder C, Grosu AL, Andratschke NH, Molls M. Proposal of human spinal cord reirradiation dose based on collection of data from 40 patients. Int J Radiat Oncol Biol Phys. 2005;61:851–5. 10.1016/j.ijrobp.2004.06.016. 15708265 10.1016/j.ijrobp.2004.06.016

[8] Nieder C, Grosu AL, Andratschke NH, Molls M. Update of human spinal cord reirradiation tolerance based on additional data from 38 patients. Int J Radiat Oncol Biol Phys. 2006;66:1446–9. 10.1016/j.ijrobp.2006.07.1383. 10.1016/j.ijrobp.2006.07.1383 17084560

[9] Kirkpatrick JP, van der Kogel AJ, Schultheiss TE. Radiation Dose-Volume Effects in the Spinal Cord. Int J Radiat Oncol Biol Phys. 2010;76 3 SUPPL. 10.1016/j.ijrobp.2009.04.095. 10.1016/j.ijrobp.2009.04.095 20171517

[10] Sahgal A, Ma L, Weinberg V, Gibbs IC, Chao S, Chang UK, et al. Reirradiation human spinal cord tolerance for stereotactic body radiotherapy. Int J Radiat Oncol Biol Phys. 2012;82:107–16. 10.1016/j.ijrobp.2010.08.021. 20951503 10.1016/j.ijrobp.2010.08.021

[11] Sahgal A, Chang JH, Ma L, Marks LB, Milano MT, Medin P, et al. Spinal cord dose tolerance to stereotactic body radiation therapy. Int J Radiat Oncol Biol Phys. 2021;110:124–36. 10.1016/j.ijrobp.2019.09.038. 31606528 10.1016/j.ijrobp.2019.09.038

[12] Iqbal MS, West N, Richmond N, Kovarik J, Gray I, Willis N, et al. Systematic Review A systematic review and practical considerations of stereotactic body radiotherapy in the treatment of head and neck cancer. 2021. https://academic.oup.com/bjr/article-abstract/94/1117/20200332/7460354?redirectedFrom=fulltext&login=false. 10.1259/bjr.20200332 PMC7774675 32960652

[13] Simone C, Amini A, Chetty I, Choi JI, Chun S, Donington J, et al. American Radium Society (ARS) and American College of Radiology (ACR) Appropriate Use Criteria Systematic Review and Guidelines on Reirradiation for Non-small Cell Lung Cancer (NSCLC). Int J Radiat Oncol Biol Phys. 2020;108(2S):E48. 10.1016/j.ijrobp.2025.03.056 PMC12874335 40185207

[14] Troost GC, Wink KCJ, Roelofs E, Simone Ii CB, Makocki S, Löck S, et al. Photons or protons for reirradiation in (non-)small cell lung cancer: results of the multicentric ROCOCO in silico study. British Journal of Radiology. 2020;93. https://academic.oup.com/bjr/article-abstract/93/1107/20190879/7449348?redirectedFrom=fulltext&login=false. 10.1259/bjr.20190879 PMC7066965 31804145

[15] Paradis KC, Mayo C, Owen D, Spratt DE, Hearn J, Rosen B, et al. The special medical physics consult process for reirradiation patients. Adv Radiat Oncol. 2019;4:559–65. 10.1016/j.adro.2019.05.007. 31681862 10.1016/j.adro.2019.05.007 PMC6817723

[16] Griffioen GHMJ, Dahele M, De Haan PF, Van de Ven PM, Slotman BJ, Senan S. High-dose, conventionally fractionated thoracic reirradiation for lung tumors. Lung Cancer. 2014;83:356–62. 10.1016/j.lungcan.2013.12.006. 24433824 10.1016/j.lungcan.2013.12.006

[17] Tetar S, Dahele M, Griffioen G, Slotman B, Senan S. High-dose conventional thoracic re-irradiation for lung cancer: updated results. Lung Cancer. 2015;88:235–6. 10.1016/j.lungcan.2015.02.008. 25736570 10.1016/j.lungcan.2015.02.008

[18] Wang HH, Chen Y, Liu X, Zaorsky NG, Mani K, Niu ZM, et al. Reirradiation with stereotactic body radiotherapy for primary or secondary lung malignancies: Tumor control probability and safety analyses. Radiotherapy and Oncology. 2023;187. 10.1016/j.radonc.2023.109817. 10.1016/j.radonc.2023.109817 37480993

[19] Gabrys D, Kulik R, Namysł-Kaletka A. Re-irradiation for intra-thoracic tumours and extra-thoracic breast cancer: dose accumulation, evaluation of efficacy and toxicity based on a literature review. British Journal of Radiology. 2022;95. 10.1259/bjr.20201292. 10.1259/bjr.20201292 PMC9153724 34826226

[20] Lee TH, Kim DY, Wu HG, Lee JH, Kim HJ. Treatment outcomes of re-irradiation using stereotactic ablative radiotherapy to lung: a propensity score matching analysis. Radiation Oncology. 2021;16. 10.1186/s13014-021-01948-6. 10.1186/s13014-021-01948-6 PMC8600824 34794471

[21] Maddalo M, D’Angelo E, Fiorica F, Argenone A, Scricciolo M, Cozzi S, et al. Thoracic re-irradiation with 3D-conformal or more advanced techniques: A systematic review of treatment safety by the Re-irradiation Study Group of the Italian Association of Radiation and Oncology AIRO. Critical Reviews in Oncology/Hematology. 2021;167. 10.1016/j.critrevonc.2021.103500. 10.1016/j.critrevonc.2021.103500 34688894

[22] Schröder C, Stiefel I, Tanadini-Lang S, Pytko I, Vu E, Guckenberger M, et al. Re-irradiation in the thorax – an analysis of efficacy and safety based on accumulated EQD2 doses. Radiother Oncol. 2020;152:56–62. 10.1016/j.radonc.2020.07.033. 32717358 10.1016/j.radonc.2020.07.033

[23] De Ruysscher D, Faivre-Finn C, Le Pechoux C, Peeters S, Belderbos J. Review High-dose re-irradiation following radical radiotherapy for non-small-cell lung cancer. 2014. https://www.thelancet.com/journals/lanonc/article/PIIS1470-2045(14)70345-6/fulltext. 10.1016/S1470-2045(14)70345-6 25456380

[24] John C, Dal Bello R, Andratschke N, Guckenberger M, Boda-Heggemann J, Gkika E, et al. In-field stereotactic body radiotherapy (SBRT) reirradiation for pulmonary malignancies as a multicentre analysis of the German Society of Radiation Oncology (DEGRO). Sci Rep. 2021;11. 10.1038/s41598-021-83210-3. 10.1038/s41598-021-83210-3 PMC7907095 33633130

[25] Rock C, Kane K, Sood S, Cao Y, Chen RC, Wang F. Reirradiation of Utracentrally Located Thoracic Tumors Using a 10-Fraction Hypofractionated Stereotactic Body Radiation Therapy Course: A Detailed Dosimetric Analysis. Adv Radiat Oncol. 2024;9. 10.1016/j.adro.2024.101626. 10.1016/j.adro.2024.101626 PMC11513450 39474010

[26] NRGOncology. RTOG 0813 Seamless phase I/II study of stereotactic lung radiotherapy (SBRT) for early stage, centrally located, non-small cell lung cancer (NSCLC) in medically inoperable patients. 2015. https://wwwnrgoncology.org/Clinical-Trials/Protocol/rtog-0813/.

[27] Giuliani M, Mathew AS, Bahig H, Bratman SV, Filion E, Glick D, et al. SUNSET: stereotactic radiation for ultracentral non–small-cell lung cancer—a safety and efficacy trial. Clin Lung Cancer. 2018;19:e529–32. 10.1016/j.cllc.2018.04.001. 29759332 10.1016/j.cllc.2018.04.001

[28] TITE-CRM Phase I Clinical Trials: Implementation Using SAS. https://sph.umich.edu/ccb/pdf/titecrm_manual_1-5-09.pdf.

[29] Brock KK, Sharpe MB, Dawson LA, Kim SM, Jaffray DA. Accuracy of finite element model-based multi-organ deformable image registration. In: Medical Physics. John Wiley and Sons Ltd; 2005. p. 1647–59. 10.1118/1.1915012. 10.1118/1.1915012 16013724

[30] Samavati N, Velec M, Brock KK. Effect of deformable registration uncertainty on lung SBRT dose accumulation. Med Phys. 2016;43:233–40. 10.1118/1.4938412. 26745916 10.1118/1.4938412 PMC4691248

[31] He Y, Cazoulat G, Wu C, Svensson S, Almodovar-Abreu L, Rigaud B, et al. Quantifying the effect of 4-dimensional computed tomography–based deformable dose accumulation on representing radiation damage for patients with locally advanced non-small cell lung cancer treated with standard-fractionated intensity-modulated radiation therapy. Int J Radiat Oncol Biol Phys. 2024;118:231–41. 10.1016/j.ijrobp.2023.07.016. 10.1016/j.ijrobp.2023.07.016 PMC11379060 37552151

[32] Cazoulat G, Owen D, Matuszak MM, Balter JM, Brock KK. Biomechanical deformable image registration of longitudinal lung CT images using vessel information. Phys Med Biol. 2016;61:4826–39. 10.1088/0031-9155/61/13/4826. 27273115 10.1088/0031-9155/61/13/4826 PMC4975156

[33] Hardcastle N, Vasquez Osorio E, Jackson A, Mayo C, Aarberg AE, Ayadi M, et al. Multi-centre evaluation of variation in cumulative dose assessment in reirradiation scenarios. Radiotherapy and Oncology. 2024;194. 10.1016/j.radonc.2024.110184. 10.1016/j.radonc.2024.110184 38453055

[34] Diez P, Hanna GG, Aitken KL, van As N, Carver A, Colaco RJ, et al. UK 2022 consensus on normal tissue dose-volume constraints for oligometastatic, primary lung and hepatocellular carcinoma stereotactic ablative radiotherapy. Clin Oncol. 2022;34:288–300. 10.1016/j.clon.2022.02.010. 10.1016/j.clon.2022.02.010 35272913

[35] Bradley JD, Paulus R, Komaki R, Masters G, Blumenschein G, Schild S, et al. Standard-dose versus high-dose conformal radiotherapy with concurrent and consolidation carboplatin plus paclitaxel with or without cetuximab for patients with stage IIIA or IIIB non-small-cell lung cancer (RTOG 0617): a randomised, two-by-two factorial phase 3 study. Lancet Oncol. 2015;16:187–99. 10.1016/S1470-2045(14)71207-0. 25601342 10.1016/S1470-2045(14)71207-0 PMC4419359

[36] Cheung YK, Chappell R. Sequential designs for phase I clinical trials with late-onset toxicities. Biometrics. 2000;56:1177–82. 10.1111/j.0006-341x.2000.01177.x. 11129476 10.1111/j.0006-341x.2000.01177.x

